# Expression Profile and Localization of SARS-CoV-2 Nonstructural Replicase Proteins in Infected Cells

**DOI:** 10.1128/spectrum.00744-22

**Published:** 2022-06-22

**Authors:** Fang-Shu Shi, Yin Yu, Ya-Li Li, Lilan Cui, Zhuangzhuang Zhao, Mi Wang, Bin Wang, Rong Zhang, Yao-Wei Huang

**Affiliations:** a Department of Veterinary Medicine, Zhejiang University, Hangzhou, China; b Key Laboratory of Medical Molecular Virology (MOE/NHC/CAMS), School of Basic Medical Sciences, Shanghai Medical College, Shanghai Institute of Infectious Disease and Biosecurity, Fudan University, China; c Novoprotein Scientific Inc., Shanghai, China; d Guangdong Laboratory for Lingnan Modern Agriculture, Guangzhou, China; Changchun Veterinary Research Institute

**Keywords:** coronavirus, SARS-CoV-2, replicase proteins, replication, subcellular localization

## Abstract

Severe acute respiratory syndrome coronavirus (SARS-CoV)-2 is responsible for the COVID-19 pandemic that has caused unprecedented loss of life and economic trouble all over the world, though the mechanism of its replication remains poorly understood. In this study, antibodies were generated and used to systematically determine the expression profile and subcellular distribution of 11 SARS-CoV-2 nonstructural replicase proteins (nsp1, nsp2, nsp3, nsp5, nsp7, nsp8, nsp9, nsp10, nsp13, nsp14, and nsp15) by Western blot and immunofluorescence assay. Nsp3, nsp5, and nsp8 were detected in perinuclear foci at different time points, with diffusion and stronger fluorescence observed over time. In particular, colocalization of nsp8 and nsp13 with different replicase proteins suggested viral protein-protein interaction, which may be key to understanding their functions and potential molecular mechanisms. Viral intermediate dsRNA was detected in perinuclear foci as early as 2-h postinfection, indicating the initiation of virus replication. With the passage of time, these perinuclear dsRNA foci became larger and brighter, and nearly all colocalized with N protein, consistent with viral growth over time. Thus, the development of these anti-nsp antibodies provides basic tools for the further study of replication and diagnosis of SARS-CoV-2.

**IMPORTANCE** The intracellular localization of SARS-CoV-2 replicase nonstructural proteins (nsp) during infection has not been fully elucidated. In this study, we systematically analyzed the expression and subcellular localization of 11 distinct viral nsp and dsRNA over time in SARS-CoV-2-infected cells by using individual antibody against these replicase proteins. The data indicated that nsp gene expression is highly regulated in space and time, which could be useful to understand the function of viral replicases and future development of diagnostics and potential antiviral strategies against SARS-CoV-2.

## INTRODUCTION

Coronaviruses (CoVs) are highly diverse RNA viruses with a broad host range; some are known to infect humans, mice, pigs, and cats among other mammalian species, causing mild to severe respiratory, intestinal and nervous system diseases (1–3). Currently, the subfamily *Coronavirinae* of the family *Coronaviridae* is classified into four genera by the International Committee on Taxonomy of Viruses: *Alphacoronavirus*, *Betacoronavirus*, *Gammacoronavirus*, and *Deltacoronavirus* (1, 4).

An unprecedented viral outbreak started in December 2019 (COVID-19) in Wuhan, Hubei Province in China (5). The most prominent clinical symptoms of the patients were dry cough, dyspnea, fever, and bilateral lung infiltration revealed by X-ray imaging, similar to those of severe acute respiratory syndrome (SARS)-CoV (6). The causative agent was named SARS-CoV-2, and according to the COVID Dashboard (https://coronavirus.jhu.edu/map.html), there have been more than 430 million confirmed cases globally, leading to at least 5.9 million deaths as of February 2022. The ongoing challenge posed by variants of SARS-CoV-2 is unprecedented, especially the “Omicron” strain, which is more contagious. In many countries, “Omicron” has become the dominant strain in circulation, including in those with high COVID-19 vaccination rates, which increases the risk of continual pandemic waves (7).

**FIG 1:**
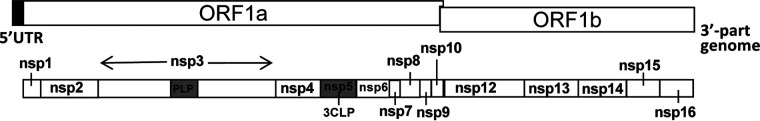
Diagram of SARS-CoV-2 replicase genes. The SARS-CoV-2 replicase is encoded by ORF1a and ORF1b, creating polyproteins which are cleaved into 16 individual nonstructural proteins (nsp).

SARS-CoV-2 is a positive-sense, single-stranded RNA virus with an ~30 kb genome that contains 12 putative open reading frames (ORFs) encoding at least 29 proteins. About two thirds of the genome consists of a large replicase (Rep) gene (ORF1a and ORF1b), followed by structural and accessory genes (5, 6). ORF1a and ORF1b encode large polyproteins, pp1a and pp1ab, respectively, that are later cleaved by viral proteases into 16 nonstructural proteins (nsp1 to 16), which participate in the transcription and replication of viral RNA (Fig. 1) (8). Ribosome analysis shows that the frameshift efficiency between ORF1a and ORF1b of SARS-CoV-2 is between 45% and 70%, similar to that of the model CoV, mouse hepatitis virus (MHV) (9, 10).

The SARS-CoV-2 replicase polyproteins are regulated by proteases encoded by two viral proteins, the 3-chymotrypsin-like protease (3CLpro or nsp5) and papain-like proteinase (PLP) within nsp3 (11). The SARS-CoV-2 nsp1 protein, located at the N-terminus of the replicase polyprotein (pp1a), has great variability between different CoVs (12, 13). Several studies reported that deletions in nsp1 and nsp2 may indicate the virus is undergoing a significant evolutionary process resulting in viral adaptation in host cells (14, 15). The nsp3 protein is the largest multidomain protein produced by CoVs, and it incorporates multifunctional domains such as an acidic domain (Ac) and PLP, among others (16). Previous studies reported that in most CoVs, the PLP protein acts as a deubiquitinase (17) and interferon antagonist (18, 19). Nsp8 has been found to be associated with RNA-dependent RNA polymerase (RdRp), and it serves as a primer for RNA synthesis (20). The nsp7 and nsp8 (nsp7-nsp8) supercomplex is an essential cofactor for the nsp12 polymerase (21). In addition, the nsp12-nsp7-nsp8 replication and transcription complex is by far the smallest complex required for nucleotide polymerization (21, 22). The CoV nsp9 protein is an RNA binding protein containing a NiRAN domain essential for CoV replication (23). The nsp13 (helicase) of SARS-CoV, encoded by ORF1b, plays a key role in catalyzing the unwinding of double-stranded oligonucleotides into single strands using the energy produced by the hydrolysis of NTPs, and the presence of viral polymerase (nsp12) can increase the helicase catalytic rate (24, 25). The nsp14 and nsp16 proteins are the mRNA cap methyltransferases, which allow CoVs to evade the human immune system (26).

In addition, the SARS-CoV-2 structural gene encodes four structural proteins, including spike glycoprotein (S protein), membrane protein (M protein), envelope protein (E protein), and nuclear protein (N protein) (5, 6), in addition to other nonessential nonstructural accessory proteins. Among the structural proteins, the N protein plays an important role in virus replication, transcription, and translation, and it also may modulate host innate immune functions (27, 28).

A number of studies on the SARS-CoV-2 replicase proteins have been published, from potential inhibitors, drug screens, structure, and function (29–32). The nsp13, nsp14, nsp15, and ORF6 proteins of SARS-CoV-2 have been certified as potent interferon antagonists (33). The distribution of nearly all SARS-CoV-2 proteins was investigated after overexpression of tagged individual viral genes in Hep-2 cells, with most detected in the cytoplasm or nucleus (34). However, the subcellular location of replicase nonstructural proteins has not been systematically determined during active SARS-CoV-2 infection by using specific anti-nsp antibodies. In the current study, specific antisera were generated against 11 SARS-CoV-2 replicase proteins (nsp1, nsp2, nsp3, nsp5, nsp7, nsp8, nsp9, nsp10, nsp13, nsp14, and nsp15, respectively) and used to track protein expression profile and distribution by Western blot (WB) and immunofluorescence assay (IFA).

## RESULTS AND DISCUSSION

### Generation and characterization of specific polyclonal antibodies against SARS-CoV-2 replicase proteins.

The sequence information of the SARS-CoV-2 strain (NC_045512.2) was used to construct expression plasmids of each individual replicase protein encoded by ORF1a and ORF1b (Fig. 1). As shown in Table 1, we created separate SARS-CoV-2 replicase constructs for nsp1, nsp2, nsp3, nsp5, nsp7, nsp8, nsp9, nsp10, nsp13, nsp14, and nsp15, placing them both in His-tagged recombinant prokaryotic vectors and Myc-tagged recombinant eukaryotic vectors. Recombinant proteins expressed from the prokaryotic expression plasmids were purified and used for production of polyclonal antisera in mice. Because the antibodies used for WB analysis of the anti-nsp4, 6, 11, 12, and 16 proteins showed high-background results, further investigation of nsp4, 6, 11, 12, and 16 proteins was not performed.

**TABLE 1:** Construction of SARS-CoV-2 nonstructural replicase genes

Protein	Cloned nucleotides	Amino acids	mol wt (kDa)	Use
nsp1	266 to 805	E2 to 180G	14	IF/IB^*a*^
nsp2	806 to 2719	A181 to 818G	75	IF/IB
nsp3 (PLP)	4955 to 5911	E1564 to 1878K	35	IF/IB
nsp5	10055 to 10972	S3264 to 3569Q	33	IF/IB
nsp7	11843 to 12091	S3860 to 3942Q	10	IF^*b*^
nsp8	12092 to 12685	A3943 to 4140Q	22	IF/IB
nsp9	12686 to 13024	N4141 to 4253Q	13	IF/IB
nsp10	13025 to 13441	A4254 to 4392Q	19	IF
nsp13	16237 to 18039	A5323 to 5923Q	67	NA^*c*^
nsp14	18040 to 19620	A5924 to 6450Q	60	IF
nsp15	19621 to 20658	S6451 to 6796Q	38	IF

a IB, immunoblot.

b IF, immunofluorescence.

c NA, not applicable.

**FIG 2:**
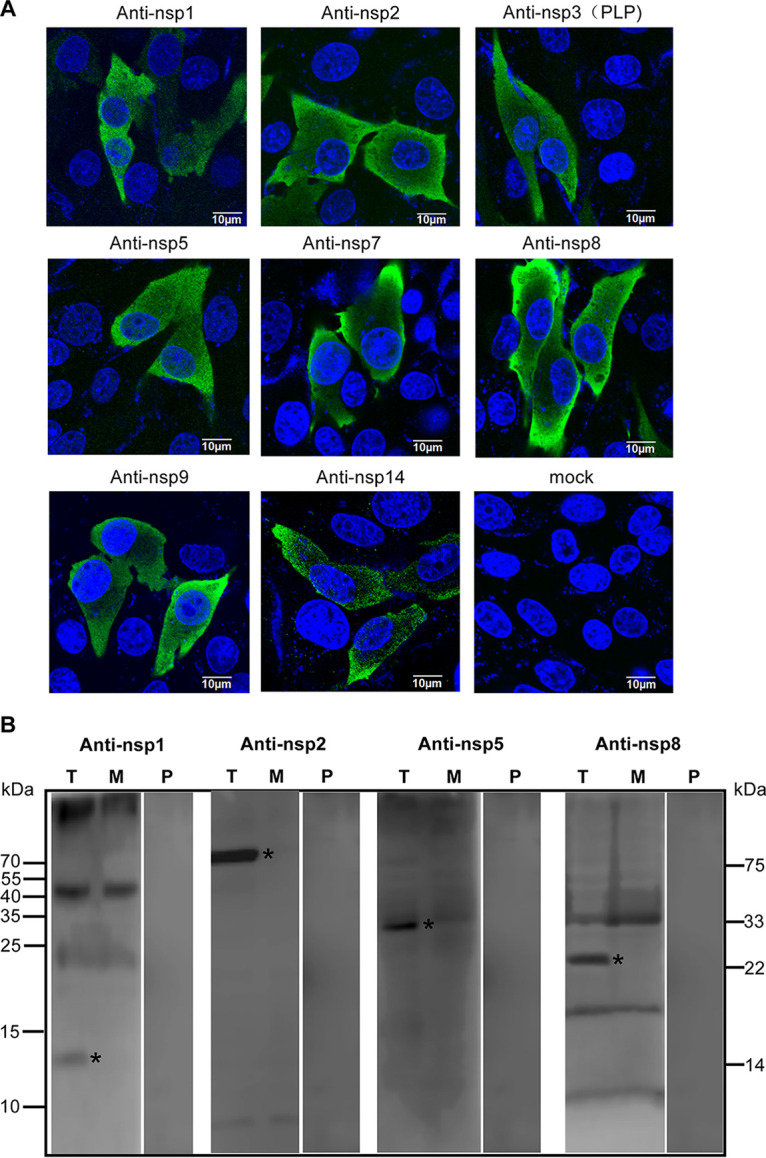
Detection of replicase proteins in BHK-21 cells by immunofluorescence and immunoblot analysis. (A) Recombinant nsp proteins were detected in transfected BHK-21 cells by mouse sera followed by secondary Alexa Fluor 488 anti-mouse IgG. (B) Western blot was performed using anti-SARS-CoV-2 serum to show recombinant replicase proteins in the transfected cells (T). Meanwhile, mock-transfected (empty PRK5 vector; M) BHK-21 cells and preimmune serum (P) were defined as control treatments.

To generate the polyclonal antisera, 90 8-week-old female BALB/c mice were immunized by intramuscular injection with recombinant nsp1, nsp2, nsp3, nsp5, nsp7, nsp8, nsp9, or nsp14 (10 mice for each protein antigen), with 10 unimmunized mice used to collect negative-control sera. A small amount of tail blood was collected for ELISA determination, and then the antisera were pooled from all mice in each group. Antiserum titers for all individual mice injected with viral nsp were greater than 1:10,000 (data not shown). Serum specificity was tested by IFA in BHK-21 cells transfected with Myc-labeled recombinant nsp proteins (pRK5-SARS-CoV-2-nsp). At 24-h posttransfection (Fig. 2A), all eight viral antigens (nsp1, nsp2, nsp3, nsp5, nsp7, nsp8, nsp9, and nsp14) were seen expressed in the cytoplasm. In addition, nsp1, nsp5, nsp9, and nsp14 were observed to be expressed in the nucleus of BHK-21 cells (Fig. 2A), similar to the pattern observed in Hep-2 cells after overexpression of tagged SARS-CoV-2 genes (34).

By WB analysis, the anti-nsp1 serum detected a discrete band of 14 kDa, while the anti-nsp2, anti-nsp5, and anti-nsp8 sera detected discrete bands at 75, 33, and 22 kDa, respectively (Fig. 2B). No staining was detected in mock-transfected (empty pRK5 vector) and in cells probed with preimmune serum (Fig. 2B).

### Western blot and immunofluorescence detection of eight SARS-CoV-2 replicase proteins during infection of cultured cells.

**FIG 3:**
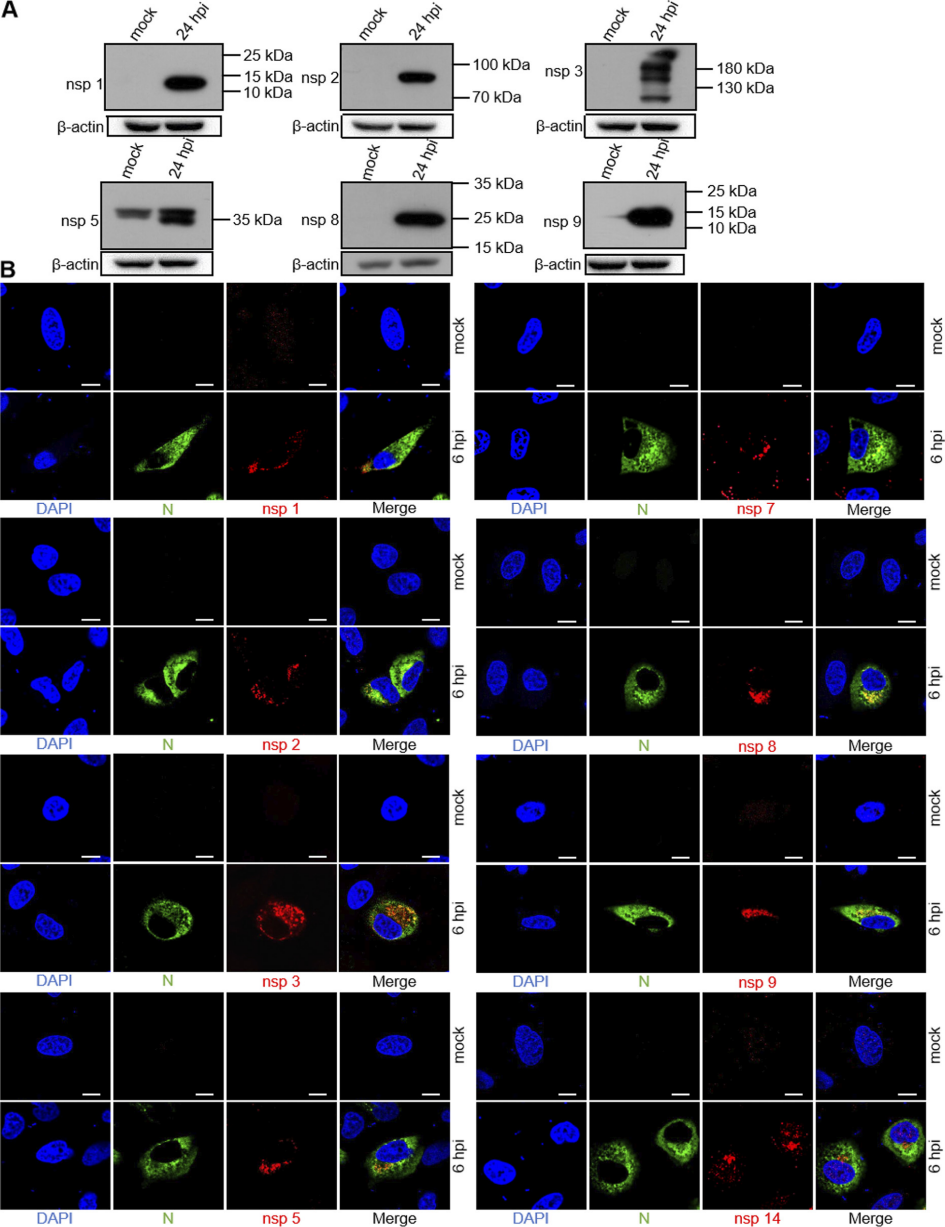
Expression and subcellular distribution of SARS-CoV-2 nonstructural proteins in A549-ACE2 cells. A549-ACE2 cells were mock infected or SARS-CoV-2-infected at an MOI of 2. (A) Cells were harvested at 24-h postinfection (hpi), total cell lysates were prepared, and proteins were separated by 10% SDS-PAGE and analyzed by Western blot with home-made mouse anti-SARS-CoV-2 nsp1, nsp2, nsp3, nsp5, nsp8, and nsp9 serum, followed by incubating with horseradish peroxidase (HRP)-conjugated goat anti-rabbit and developed using SuperSignal West Pico chemiluminescent substrate. Numbers on the right indicate protein sizes in kDa. (B) At 6 hpi, the cells were fixed, permeabilized, and costained with rabbit anti-SARS nucleocapsid (N) protein polyclonal antibody (pAb) and the appropriate home-made mouse anti-SARS-CoV-2 nsp sera, followed by staining with Alexa Fluor 488 conjugated goat anti-rabbit Ab (green) and Alexa Fluor 555 conjugated goat anti-mouse Ab (red). Cell nuclei were stained with DAPI (blue) and examined by confocal microscopy; scale bars represent 10 μm.

To characterize the expression of SARS-CoV-2 replicase proteins during infection of cultured A549-ACE2 cells, we used the polyclonal antisera generated above to detect at 24-h postinfection (hpi). The anti-nsp1 serum detected a single protein band with an observed mass of 14 kDa, consistent with the predicted nsp1 protein cleaved at 180G↓A181 (Fig. 3A). The anti-nsp2 serum predominantly detected a protein at 75 kDa, consistent with the predicted nsp2 cleaved at 180G↓A181 and 818G↓A819 (Fig. 3A). The anti-nsp2 serum also detected nonspecific weak bands at 130 kDa and 40 to 55 kDa (not shown), which were also present in the mock-infected cells. The anti-nsp3 serum detected several bands between 100 and 130 kDa and above in the infected-cell lysates that were not detected in mock-infected cells (Fig. 3A). The anti-nsp5 serum detected a 33 kDa protein in infected-cell lysates, consistent with the predicted nsp5 protein cleaved at 3263Q↓S3264 and 3569Q↓S3570 (Fig. 3A), as well as a nonspecific band between 40 and 55 kDa. The anti-nsp8 serum detected a protein of 21 kDa from infected-cell lysates, consistent with the predicted nsp8 protein cleaved at 3942Q↓A3943 and 4140Q↓N4141 (Fig. 3A). No immunoreactivity was detected from mock-infected lysates for the anti-nsp8 serum, clearly demonstrating the specificity of the immune serum. The anti-nsp9 serum detected a protein of 12 kDa, consistent with the predicted nsp9 protein cleaved at 4140Q↓N4141 and 4253Q↓A4254 (Fig. 3A).

The CoV N protein encapsidates viral genome RNA, playing a vital role in the viral life cycle, and is abundantly expressed during infection (35). Earlier studies demonstrated that the SARS-CoV N protein was detectable in small cytoplasmic foci in infected cells as early as 3 hpi (36). We thus used the N protein as a detecting marker for viral replication in cells in the confocal microscopy experiments. In addition, one study demonstrated that most SARS-CoV replicase proteins could be detected by specific nsp antibodies as early as 6 hpi (11). To determine the intracellular localization of SARS-CoV-2 nsp proteins, A549-ACE2 cells were mock infected or infected with SARS-CoV-2 for 6 h, and then an IFA was carried out using N protein- and nsp-specific serum (Fig. 3B). According to confocal microscopy, the nsp1 signal was located in punctate perinuclear and cytoplasmic foci at 6 hpi (Fig. 3B), similar to the pattern of nsp1 antibody observed in SARS-CoV-infected Vero cells (11). Nsp2 was also detected in perinuclear foci and colocalized with N protein (Fig. 3B), suggesting that nsp2 is found at the site of viral RNA replication.

Our previous study indicated that nsp3 of swine acute diarrhea syndrome (SADS)-CoV, an alphacoronavirus, was detected in perinuclear foci at 4 hpi (37). Stertz et al. also demonstrated a clear colocalization of SARS-CoV nsp3 and N in cytoplasmic foci in infected Vero cells at 3 hpi (36). As expected, we observed colocalization of nsp3 and N in SARS-CoV-2 infected A549-ACE2 cells at 6 hpi (Fig. 3B). The nsp3 signal seemed hollow and displayed a higher fluorescence intensity, similar to the foci reported for SARS-CoV nsp3 (36). It has been shown how cytoplasmic foci of SARS-CoV nsp1, nsp2, nsp3, nsp5, nsp8, nsp9, and nsp13 proteins were distributed in Vero cells (11). Similarly, as early as 6 hpi, SARS-CoV-2 nsp5, nsp7, nsp8, and nsp9 were detectable in small cytoplasmic and punctate perinuclear foci.

SARS-CoV-2 is an RNA virus that replicates its genome, translates proteins, and assembles progeny viral particles in the cytoplasm. It depends on the infrastructure of the host cell and metabolism for nearly all stages of its replication cycle. Release of the viral genome into the cytoplasm of host cells begins a complex program of viral gene expression, which is highly regulated in space and time (38, 39). However, previous studies demonstrated that some viral proteins are also detectable in the nucleus, including nsp1, nsp5, nsp9, nsp13, nsp14, and nsp16 in Hep-2 cells after overexpression of tagged individual viral genes (34). In this study, in addition to the anti-nsp14 signal located in the perinuclear and cytoplasmic foci, there was also fluorescence in the nucleus of SARS-CoV-2 infected A549-ACE2 cells at 6 hpi (Fig. 3B), suggesting in a difference during the process of replication for nsp14.

### Time-course study of nsp3, nsp5, and nsp8 localization in SARS-CoV-2-infected cells.

**FIG 4:**
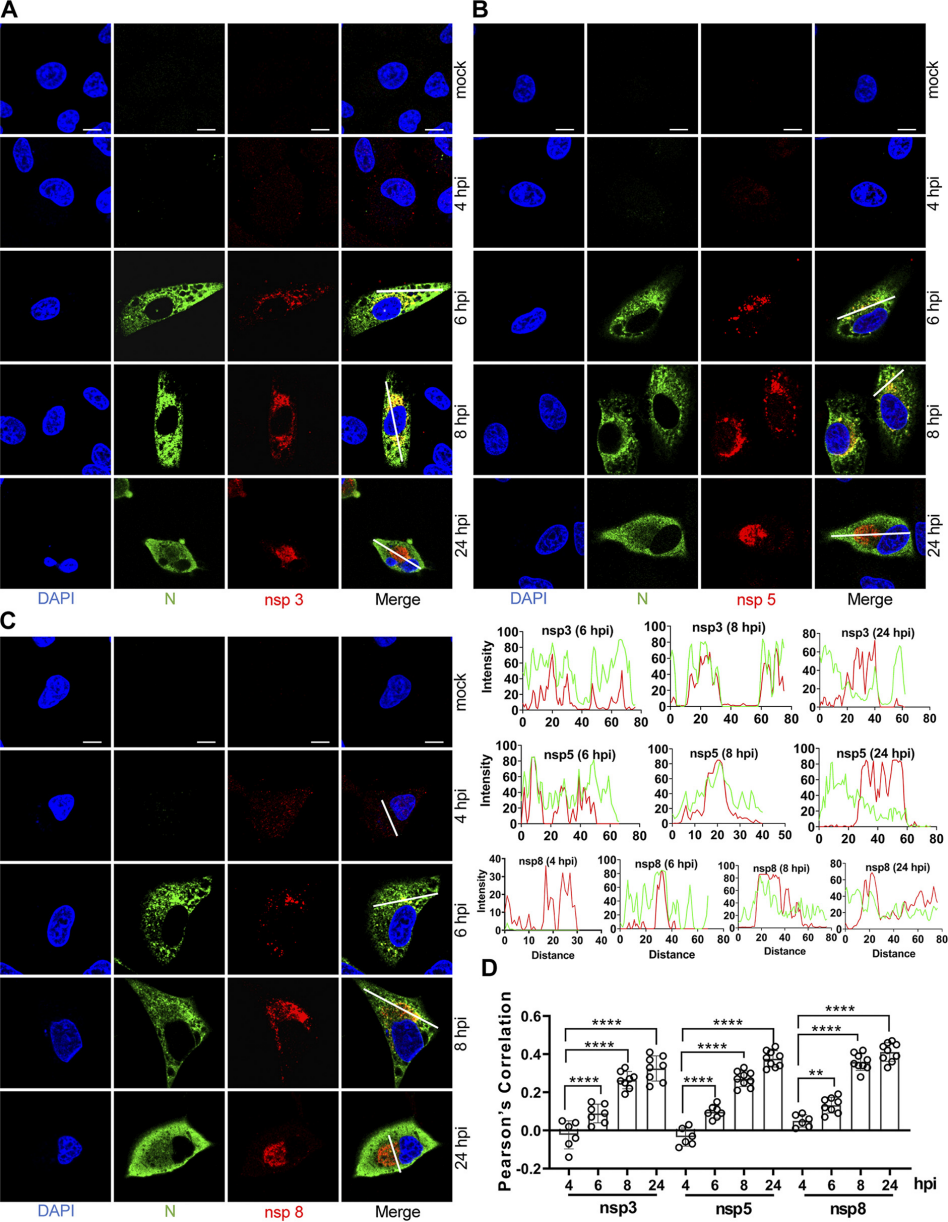
Time course of nsp3, nsp5, and nsp8 detection. A549-ACE2 cells were mock infected or infected with SARS-CoV-2 (MOI = 2), fixed at 2-, 3-, 4-, 6-, 8-, and 24-h postinfection (hpi), and costained with rabbit anti-SARS nucleocapsid (N) protein pAb and home-made mouse anti-SARS-CoV-2 nsp3 (A), nsp5 (B), or nsp8 (C). Staining was carried out with Alexa Fluor 488 conjugated goat anti-rabbit Ab (green) and Alexa Fluor 555 conjugated goat anti-mouse Ab (red). Cell nuclei were stained with DAPI (blue) and examined by confocal microscopy; scale bars represent 10 μm. The intensity distribution describes the timing of expression of nsp3, nsp5 or nsp8 for specific fluorescence along the indicated line. (D) Pearson’s correlation was used to analyze the changes in nsp3, nsp5, or nsp8 over time. One-way analysis of variance (ANOVA) was used for multiple comparisons between different times among the nsp8 or nsp13 in GraphPad Prism 8.4.3 software. ****, *P* < 0.0001; **, *P* < 0.01.

To determine the intracellular localization and timing of expression of the viral replicase proteins in greater detail, a confocal time course study of nsp3, nsp5, and nsp8 was performed. These three proteins were chosen because the observed staining intensity with the prepared antibodies was higher than that of the others tested (Fig. 3B). A549-ACE2 cells on glass coverslips were infected with SARS-CoV-2, fixed at 2, 3, 4, 6, 8, and 24 hpi, and costained with rabbit anti-SARS-CoV N and mouse anti-SARS-CoV-2 nsp3, nsp5, or nsp8 (Fig. 4). The nsp3-stained structures grew larger as time progressed, with the hollow centers being first visible at 6 hpi (Fig. 4A, D), probably reflecting that nsp3 is associated with sites of viral RNA replication in the early infection phase. Nsp5 was detected in perinuclear foci at 6 hpi, with diffusion and stronger fluorescence observed over time (Fig. 4B, D). The timing of the higher fluorescence intensity and colocalization with N protein suggests interaction with the viral replication-transcription complex.

It has been shown that point mutations in nsp7 and nsp8 of SARS-CoV delayed viral growth, identifying those residues as critical for viral replication (40). Discrete fluorescent signal was detected throughout the cytoplasm by the anti-nsp8 serum as early as 4 hpi, after which the fluorescent signal gradually increased and concentrated around the nucleus at 6 and 8 hpi, finally gathering into a perinuclear clump at 24 hpi (Fig. 4C, D). These nsp8-positive foci are expected to be the sites of early replication. No specific signals were observed at 2 and 3 hpi for nsp3, nsp5, and nsp8 (data not shown).

### Detection of dsRNA in SARS-CoV-2-infected cells.

**FIG 5:**
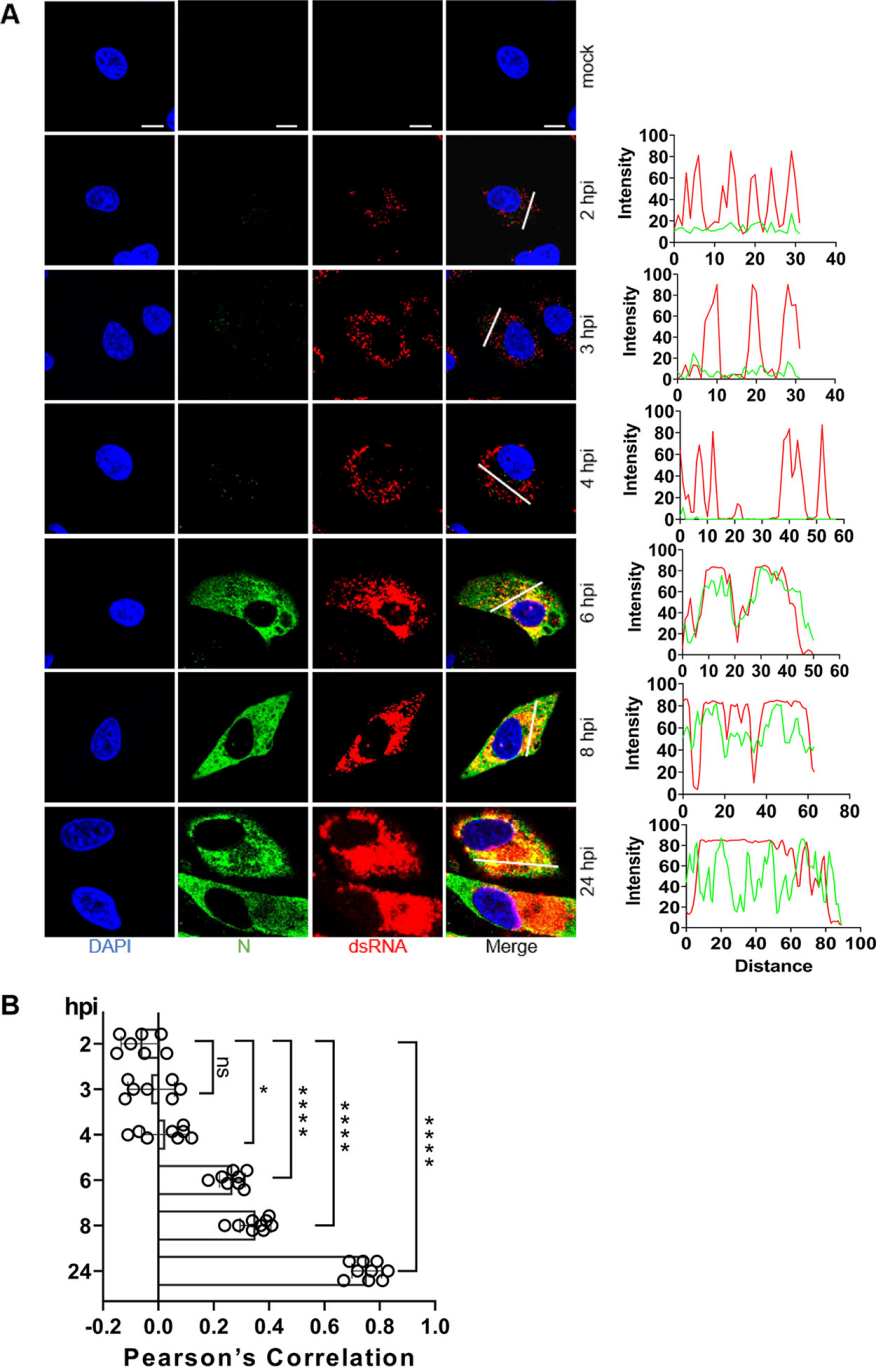
Detection of dsRNA in SARS-CoV-2-infected cells. (A) A549-ACE2 cells were mock infected or infected with SARS-CoV-2 (MOI = 2), fixed at 2-, 3-, 4-, 6-, 8-, and 24-h postinfection (hpi), and costained with rabbit anti-SARS nucleocapsid (N) protein pAb and mouse anti-dsRNA monoclonal antibody (MAb). Staining was carried out with Alexa Fluor 488-conjugated goat anti-rabbit Ab (green) and Alexa Fluor 555-conjugated goat anti-mouse Ab (red). Cell nuclei were stained with DAPI (blue) and examined by confocal microscopy; scale bars represent 10 μm. The intensity distribution describes the timing of expression of the viral RNA and N proteins for specific fluorescence along the indicated line. (B) Pearson’s correlation was used to analyze changes to dsRNA and N over time. One-way analysis of variance (ANOVA) was used for multiple comparisons between different times among the viral proteins in GraphPad Prism 8.4.3 software. ****, *P* < 0.0001; *, *P* < 0.1; ns, no significance.

CoVs produce double-stranded RNA (dsRNA) intermediates during the process of replication (41), and they have been visualized for SARS-CoV-2 and other RNA viruses by using specific antibodies *in situ* (42, 43). Such dsRNA intermediates are usually associated with replication factories found in “double-membrane vesicles” (DMVs) during infection (38). To determine the intracellular localization and timing of expression of the viral RNA in greater detail, a confocal time course study of dsRNA was performed. dsRNA was detected in perinuclear foci at 2 hpi, while mock-infected cells were essentially devoid of signal (Fig. 5), similar to that observed in SARS-CoV-infected Vero-E6 cells (38). Given that the formation of dsRNA depends on viral replicase proteins, this is direct evidence that some replicase proteins must have been produced as early as 2 hpi. With the passage of time, the perinuclear dsRNA foci became larger and brighter, and nearly all colocalized with N protein, showing the viral growth over time and demonstrating that SARS-CoV-2 replicates surprisingly fast (Fig. 5A, B).

### SARS-CoV-2 replicase proteins colocalize with each other and with a marker for dsRNA.

**FIG 6:**
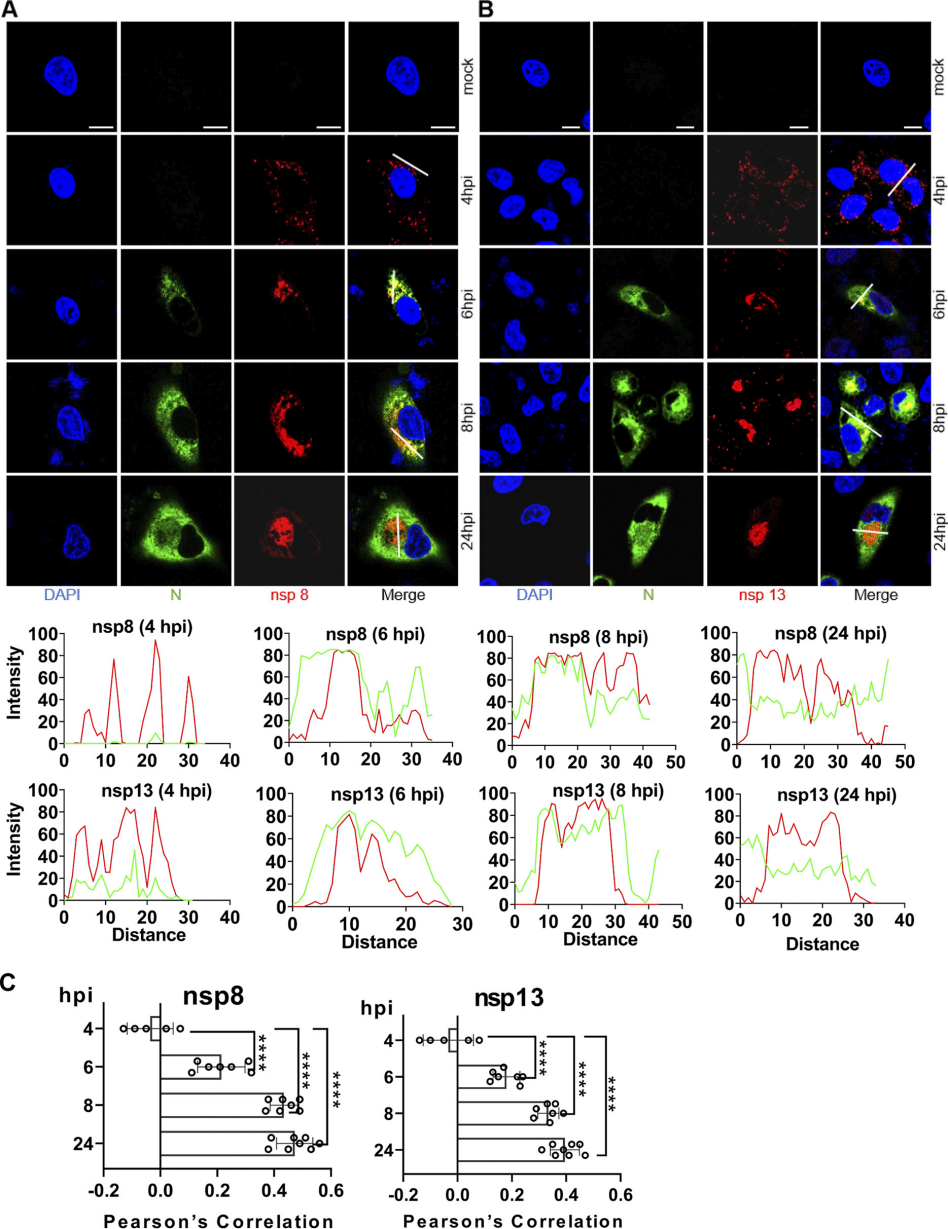
Time course of nsp8 and nsp13 detection. A549-ACE2 cells were mock infected or infected with SARS-CoV-2 (MOI 2), fixed at 2-, 3-, 4-, 6-, 8-, and 24-h postinfection (hpi), and costained with home-made mouse anti-SARS-CoV-2 N protein serum and rabbit anti-SARS nsp8 (A) or nsp13 (B) polyclonal, followed by staining with goat anti-mouse secondary antibody conjugated with Alexa Fluor 488 (green) and goat anti-rabbit secondary antibody conjugated with Alexa Fluor 555 (red). The cell nuclei were stained with DAPI (blue) and examined by confocal microscopy. No specific signal was observed at 2 and 3 hpi (not shown); scale bars represent 10 μm. The intensity distribution describes the timing of expression of nsp8 or nsp13 for specific fluorescence along the indicated line. (C) Pearson’s correlation was used to analyze changes in nsp8 or nsp13 over time. One-way analysis of variance (ANOVA) was used for multiple comparisons between different times among the nsp8 or nsp13 in GraphPad Prism 8.4.3 software. ****, *P* < 0.0001.

Analysis of viral protein-protein interaction is an important step to understand the function of viral proteins and their potential molecular mechanisms. Nsp8 has been found to be associated with RdRp and its function is defined as a primer for RNA synthesis (20, 44). Nsp13 is a helicase, which may play a role in mRNA capping and is the likely site of CoV RNA synthesis (45). Therefore, we used commercial rabbit anti-nsp8 and anti-nsp13 antibodies to determine the intracellular localization and timing of expression of the viral replicase proteins in greater detail. Like our home-made mouse anti-nsp8 sera, the commercial rabbit anti-nsp8 polyclonal showed a signal as early as 4 hpi, and it gradually increased and concentrated around the nucleus over time (Fig. 6A, C). The slight discrete cytoplasmic foci were first detected by the commercial rabbit anti-nsp13 at 4 hpi, and the fluorescent signal became more intense by 24 hpi (Fig. 6B, C), suggesting a change in the distribution of nsp13 over time.

**FIG 7:**
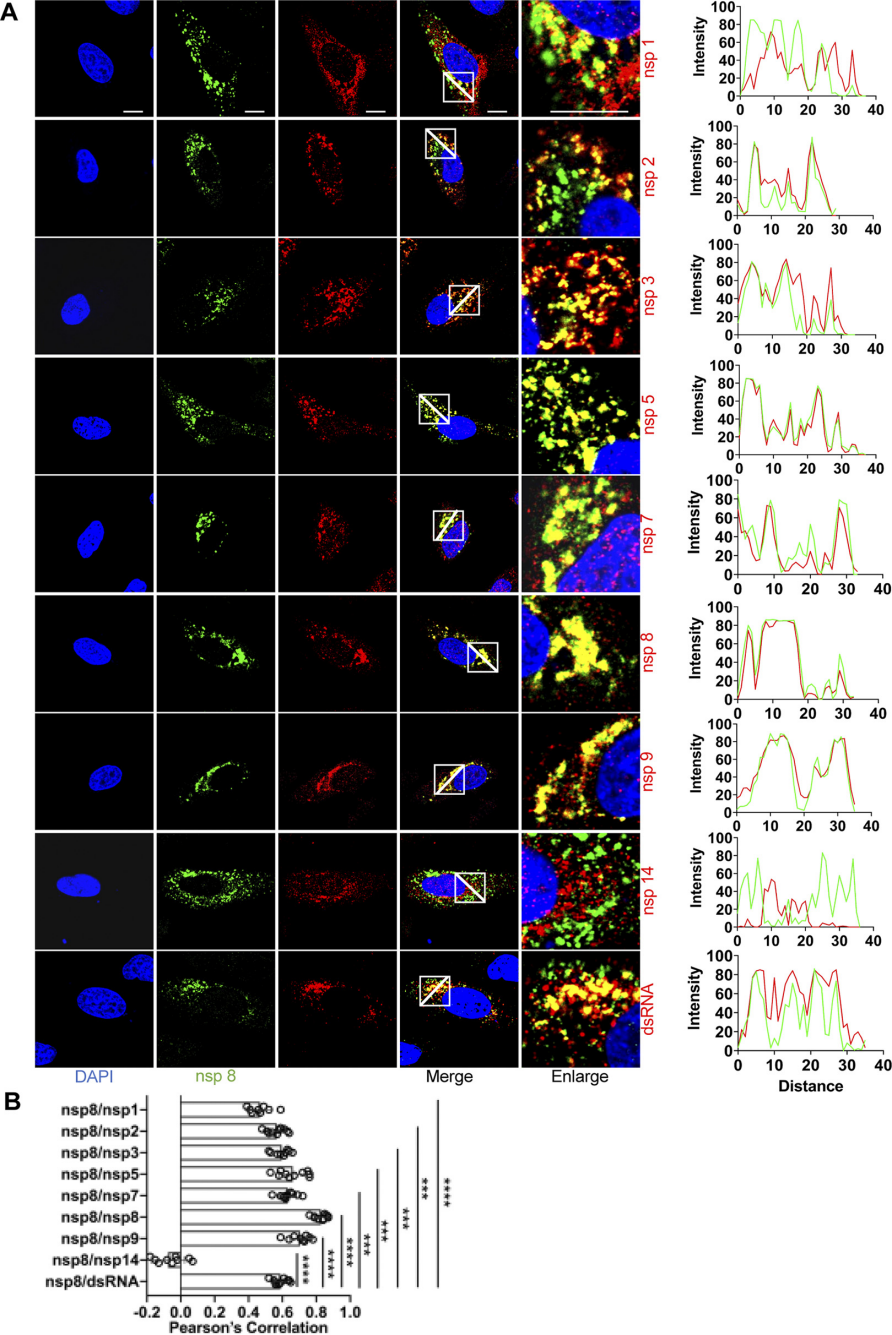
Colocalization of nsp8 with other replicase proteins and dsRNA in SARS-CoV-2-infected cells. (A) A549-ACE2 cells were infected with SARS-CoV-2 (MOI = 2), fixed at 6-h postinfection (hpi), and costained with rabbit anti-SARS nsp8 and appropriate home-made mouse anti-SARS-CoV-2 nsp sera or mouse anti-dsRNA MAb, followed by staining with Alexa Fluor 488-conjugated goat anti-rabbit (green) and Alexa Fluor 555-conjugated goat anti-mouse (red). Cell nuclei were stained with DAPI (blue) and examined by confocal microscopy. Images in the fifth column were obtained at higher magnification to show single-cell details of fluorescence labeling; scale bars represent 10 μm. The intensity distribution describes the colocalization of nsp8 with other replicase proteins for specific fluorescence along the indicated line. (B) Pearson’s correlation analysis demonstrated colocalization of nsp8 with other replicase proteins. One-way analysis of variance (ANOVA) was used for multiple comparisons on the colocalization between nsp8 and different nsps in GraphPad Prism 8.4.3 software. ****, *P* < 0.0001; ***, *P* < 0.001.

Moreover, we analyzed the colocalization between different SARS-CoV-2 proteins using rabbit anti-nsp8 antibody and home-made mouse immune sera against the other replicase proteins. Dual-labeling experiments were carried out in A549-ACE2 cells at 6 hpi (Fig. 7). We found a clear punctate colocalization of nsp8 and nsp1 in cytoplasmic foci at 6 hpi. Earlier studies demonstrated that the nsp7 and nsp8 proteins could play important roles in CoV RNA synthesis (46). In addition, the interaction between nsp7, nsp8, and nsp12 is conserved among SARS-CoV and SARS-CoV-2 (47). Colocalization of nsp8 with nsp2, nsp3, nsp5, nsp7, or nsp9 supports that these nsp proteins actually represent the same RNA transcription and replication complex that binds different replicase antibodies. However, there was little colocalization of nsp8 and nsp14 in SARS-CoV-2-infected cells where specific signal was detected (Fig. 7).

**FIG 8:**
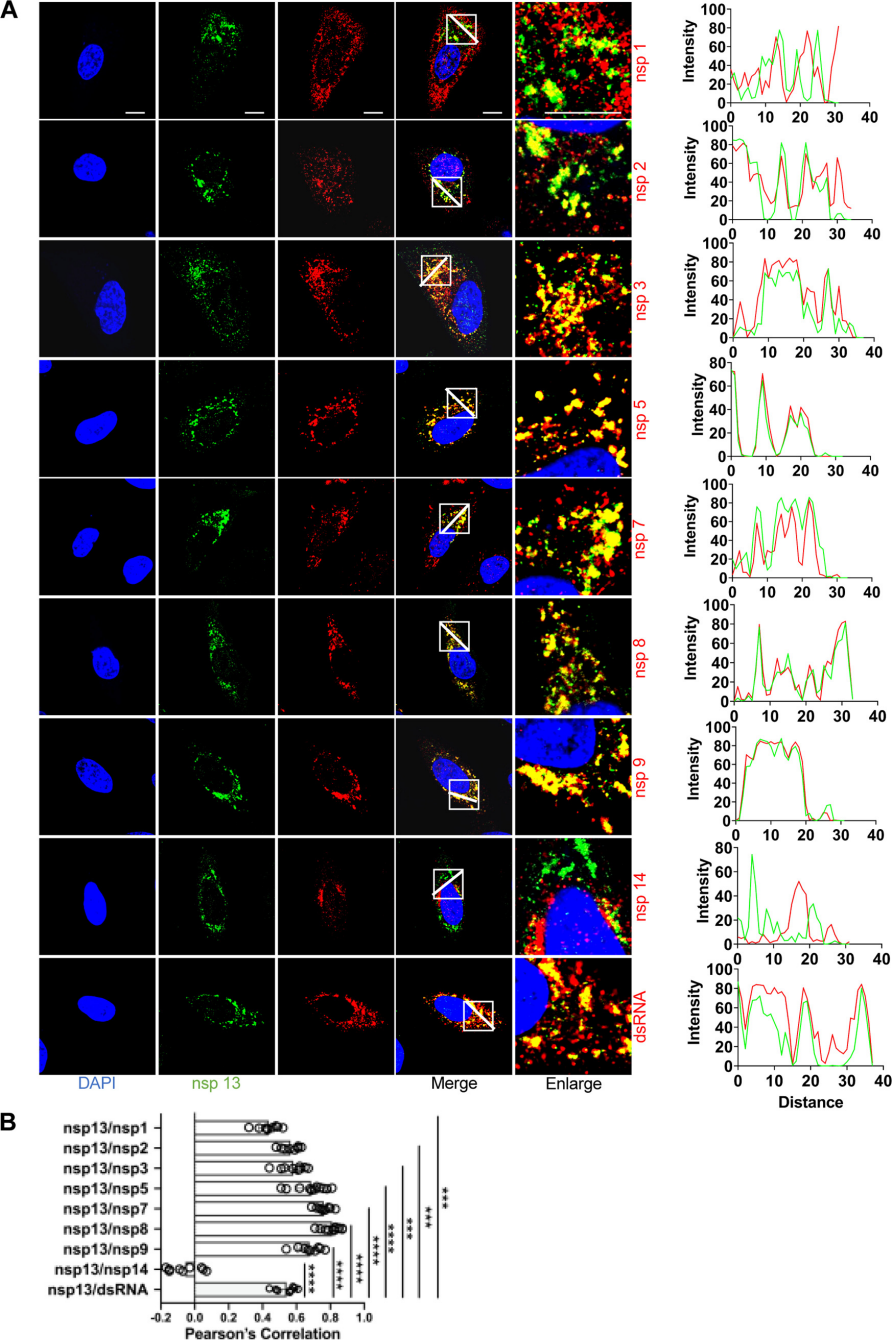
Colocalization of nsp13 with other replicase proteins and dsRNA in SARS-CoV-2-infected cells. (A) A549-ACE2 cells were infected with SARS-CoV-2 (MOI = 2), fixed at 6-h postinfection (hpi), and costained with rabbit anti-SARS nsp13 pAb and appropriate home-made mouse anti-SARS-CoV-2 nsp sera or mouse anti-dsRNA MAb, followed by staining with Alexa Fluor 488-conjugated goat anti-rabbit Ab (green) and Alexa Fluor 555-conjugated goat anti-mouse Ab (red). Cell nuclei were stained with DAPI (blue) and examined by confocal microscopy. Images in the fifth column were obtained at higher magnification to show single-cell details of fluorescence labeling; scale bars represent 10 μm. The intensity distribution describes the colocalization of nsp13 with other replicase proteins for specific fluorescence along the indicated line. (B) Pearson’s correlation analysis demonstrated colocalization of nsp13 with other replicase proteins. One-way analysis of variance (ANOVA) was used for multiple comparisons on the colocalization between nsp13 and different nsps in GraphPad Prism 8.4.3 software. ****, *P* < 0.0001; ***, *P* < 0.001.

The nsp13 helicase has been identified as an ideal target for antiviral drug development because of its conserved sequence and its indispensability in all CoVs (48, 49). We analyzed colocalization of nsp13 with the other nsps by using commercial rabbit anti-nsp13 (Fig. 8). To a certain extent, nsp13 and nsp1, nsp2, nsp3, nsp5, nsp7, nsp8, or nsp9 showed punctate colocalization, suggesting that a biologically meaningful interaction occurs between them. Meanwhile, the dsRNA also showed nearly complete colocalization with both nsp8 and nsp13 in the punctate foci. Notably, the colocalization between nsp14 and nsp13 was little, possibly suggesting a different protein-protein interaction. In addition, the perinuclear foci of nsp13 became larger and brighter, and colocalized with nearly all of the nsp tested except for nsp14, supporting its multifunctional role during viral infection (Fig. 8).

### Interaction between SARS-CoV-2 nsp8/nsp13 and other replicase proteins.

**FIG 9:**
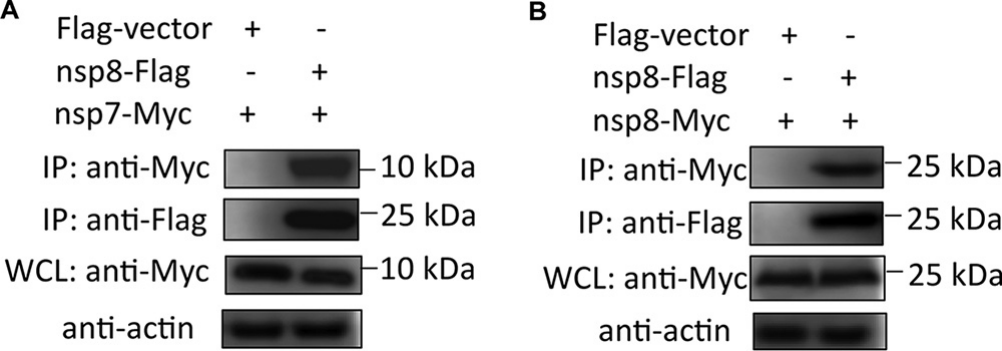
Interaction of nsp8 with other replicase proteins. HEK-293T cells were transfected with equal amounts (2 μg) of the empty vector pRK5-Flag alone (as a control), pRK5-nsp8-Flag, and pRK5-nsp7 (A)/nsp8-Myc (B). Whole-cell lysates (WCLs) were pre-adsorbed onto Dynabeads protein G and incubated with anti-Flag antibodies for coimmunoprecipitation analysis. The protein complex was detected by a horseradish peroxidase (HRP)-conjugated anti-Flag MAb and HRP-conjugated anti-Myc MAb.

Several studies have found that the nsp7 and nsp8 (nsp7-nsp8) supercomplex are essential cofactors for nsp12 polymerase, which revealed the structural basis for RNA replication (50). In addition, several studies reported the structure of nsp7-8-12-13 in complex with RNA (51). To validate the physical interactions between nsp8/nsp13 and others, a coimmunoprecipitation assays (co-IP) was conducted. HEK-293T cells were cotransfected with SARS-CoV-2-nsp8/nsp13-Flag and some nsp-Myc plasmids, including nsp1, nsp2, nsp3, nsp5, nsp7, nsp8, nsp9, and nsp14, and transfection with empty vector was used as control (Fig. 9). The results showed that nsp8-Flag coprecipitated with nsp7-Myc and nsp8-Myc, suggesting nsp7 and nsp8 indeed interact (Fig. 9). Meanwhile, Jingjiao Li et al. also demonstrated that SARS-CoV-2 intraviral nsp8 and nsp7/8 protein-protein interactions by co-IP (52), which is consistent with the protein-protein interactions seen in our study. Although SARS-CoV-2 nsp8 colocalized with nsp1, nsp2, nsp3, nsp5, nsp7, nsp8, and nsp9 (Fig. 7), we only found a physical interaction between nsp8 and nsp7 (Fig. 7), suggesting nsp8 is regulation in space and time with others. Similarity, there was no evidence of interaction between SARS-CoV-2 nsp13 and the other replicase proteins analyzed by co-IP (nsp1, nsp2, nsp3, nsp5, nsp7, nsp8, nsp9, and nsp14; data not shown).

In summary, we systematically analyzed the expression profile and localization of nsp1, nsp2, nsp3, nsp5, nsp7, nsp8, nsp9, and nsp14 proteins and dynamic changes of nsp3, nsp5, nsp8, and nsp13 proteins over time. Our data demonstrate that viral nsp gene expression is highly regulated in space and time, and these details may be helpful for understanding the function of viral replicases and future development of diagnostics, potential antiviral strategies, and nsp-antigen-based vaccines against SARS-CoV-2.

## MATERIALS AND METHODS

### Cells and viruses.

BHK-21 cells and HEK-293T cells were cultured in Dulbecco’s modified Eagle medium (DMEM; HyClone) supplemented with 10% fetal bovine serum (FBS; HyClone) and 1% (wt/vol) antibiotics (penicillin and streptomycin; HyClone). A549 cells (ATCC #CCL-185) were cultured in DMEM supplemented with 10% fetal bovine serum (FBS, Gemini), 1% penicillin and streptomycin (HyClone), 1x nonessential amino acids (HyClone), 10 mM 4-(2-hydroxyethyl)-1-piperazine ethanesulfonic acid (HEPES, HyClone), and 2 mM l-glutamine (Gibco). The A549-ACE2 clonal cell lines were generated by lentiviral transduction of the human ACE2 gene. All cells were grown at 37°C with 5% CO_2_. The SARS-CoV-2 nCoV-SH01 strain (GenBank accession no. MT121215) clone Sdel was propagated in Vero E6 cells in the absence of trypsin, and the resulting infectious virus stock used in this study had a 21-nt deletion in the spike gene (53). All infectious SARS-CoV-2-related experiments were performed in the biosafety level 3 (BSL-3) laboratory of Fudan University.

### Plasmid design and protein expression.

To express the SARS-CoV-2 replicase proteins (NC_045512.2), which is made up of nsp1, nsp2, nsp3, nsp5, nsp7, nsp8, nsp9, nsp10, nsp13, nsp14, and nsp15, gene fragment were cloned into the prokaryotic expression vector pET-30a with an N/C-terminal His-tag, yielding plasmids pET-30a-SARS-CoV-2-nsps. These plasmids were transformed into Escherichia coli BL21(DE3) cells, and His-tagged proteins were purified using Ni-NTA His-Tagged (TransGen Biotech) according to the manufacturer’s protocol. Similarly, replicase gene fragments were also cloned into the eukaryotic expression vector pRK5 with a C-terminal Myc tag (pRK5-SARS-CoV-2-nsps) for detection by antisera. Similarly, replicase gene fragments were also cloned into the eukaryotic expression vector pRK5 with an N/C-terminal Myc or Flag tag (pRK5-SARS-CoV-2-nsps) for detection by antisera.

### Antisera preparation.

Eight-week-old female BALB/c mice were immunized by intramuscular injection with 15 μg of purified protein that was mixed equally with mouse rapid immune adjuvant (Biodragon, KX0210041). After 3 weeks, the mice were immunized with the same dose, and blood samples were collected about 2 weeks after the second immunization.

### Immunofluorescence assay.

The recombinant eukaryotic plasmids were transfected into BHK-21 cells grown in 24-well plates to 70% to 80% confluence using Lipofectamine 3000 (Thermo Fisher Scientific) according to the manufacturer’s protocol. After 24 h, the medium was removed and wells were washed twice with phosphate-buffered saline (PBS), then fixed with 4% paraformaldehyde (PFA) for about 15 min at room temperature (RT). After washing three times with PBS for 3 min, cells were permeabilized in 0.5% Triton X-100 for 10 min at RT and washed with PBS repeatedly, then incubated with the primary antibody (mouse serums) diluted in 3% bovine serum albumin (BSA)/PBS for 1 h at 37°C. After washing with PBS, the samples were incubated with secondary antibody diluted in PBS for 1 h at 37°C, washed with PBS, and stained with 4’,6-diamidino-2-phenylindole (DAPI) for about 5 min at RT. For the staining of virus-infected cells, A549-ACE2 cells grown on 12-mm coverslips in a 24-well plate were infected with SARS-CoV-2 at an MOI of 2 as described previously (53). Immunofluorescence staining was performed after fixation with 2% PFA in PBS for 15 min at RT and permeabilized in 0.2% Triton X-100 in PBS for 15 min at RT. Cells were blocked for 1 h with 5% BSA and 0.3 M glycine in PBS, then incubated with primary antibody diluted in 1% BSA/PBS overnight at 4°C. After washing with PBS, secondary antibody diluted in 1% BSA/PBS was added for 1 h at RT, then cells were counterstained with DAPI for 5 min at RT.

The following home-made mouse antibodies (and dilutions) were used: anti-nsp1 (1:200); anti-nsp2 (1:200); anti-nsp3 (1:200); anti-nsp5 (1:200); anti-nsp7 (1:200); anti-nsp8 (1:200); anti-nsp9 (1:200); anti-nsp14 (1:200); anti-nucleocapsid (N) protein (1:1,000).

The following commercial antibodies were used in the study: mouse anti-dsRNA monoclonal antibody J2 (SCICONS #10010200, 1:1,000); rabbit anti-SARS nsp8 (Rockland #100-401-A53, 1:600); rabbit anti-SARS nsp13 (Rockland #100-401-A54, 1:600); rabbit anti-SARS N protein (Rockland #200-401-A50, 1:1,000); goat anti-mouse antibody conjugated with Alexa Fluor 488 (Thermo #A-28175, 1:1,000); goat anti-mouse antibody conjugated with Alexa Fluor 555 (Thermo #A-21424, 1:1,000); goat anti-rabbit antibody conjugated with Alexa Fluor 488 (Thermo #A-11034, 1:1,000); and goat anti-rabbit antibody conjugated with Alexa Fluor 555 (Thermo #A-32732, 1:1,000).

### Confocal microscopy.

Confocal fluorescent images were obtained by confocal laser scanning microscopy (CLSM, Leica TCS SP8) using an inverted oil immersion lens with a 63x objective. DAPI was excited with a 405-nm diode laser, Alexa Fluor 488 with a 488-nm argon laser, and Alexa Fluor 555 with a 561-nm DPSS laser. For fluorescence detection, respective emission filters were used, and images were analyzed by Leica LAS X system software. All immunofluorescence assays were performed at least three times.

### Western blot and co-IP.

Transfected cells were lysed in lysis buffer (1 mM Na_3_VO_4_, 10 mM NaF, 25 mM Tris-HCl, 200 mM NaCl, 25 mM β-glycerophosphate, 1% NP40 and protease cocktail [Biotool, Houston, TX]) at 4°C for 15 min, centrifuged 12,000 × *g* for 15 min at 4°C, and collected for SDS-PAGE or WB analysis. For co-IP, whole-cell lysates (WCLs) were preadsorbed onto Dynabeads protein G (Thermo Fisher Scientific) and incubated with anti-Flag antibodies in order to precipitate Flag-tagged proteins for 12 h at 4°C on a rotator. After washing with PBST three times, the resins were eluted with elution buffer (50 mM glycine, pH 2.8) for WB. Samples were transferred onto a polyvinylidene difluoride (PVDF) membrane that was subsequently blocked with Tris-buffered saline and Tween 20 (TBST) containing 5% nonfat powdered milk (Sangon Biotech) for about 2 h at RT. After washing with TBST three times, the primary antibody (mouse serums) was added and incubated overnight at 4°C. After washing with TBST, the horseradish peroxidase (HRP)-conjugated secondary antibodies (Thermo Fisher Scientific) was incubated with the membrane for 1 h. Finally, the samples of proteins were detected using an ECL kit (FDbio science).

For the viral infection experiments, virus-infected cells were lysed in RIPA buffer (Cell Signaling #9806S) containing protease inhibitors (Sigma-Aldrich # S8830), and proteins were separated via 10% SDS-PAGE and transferred to PVDF membranes. Membranes were blocked with 5% skim milk (BD Difco #232100) in TBST for 1 h at RT, and probed with the primary antibodies at 4°C overnight. After washing with TBST, blots were incubated with horseradish peroxidase (HRP)-conjugated secondary antibodies for 1 h at RT. Then blots were washed again with TBST, and developed using SuperSignal West Pico PLUS Chemiluminescent Substrate (Thermo Fisher #34577).

The following home-made antibodies (and dilutions) were used: anti-nsp1 (1:500); anti-nsp2 (1:500); anti-nsp3 (1:500); anti-nsp5 (1:500); anti-nsp8 (1:500); anti-nsp9 (1:500). The following commercial antibodies were used: rabbit anti-β-actin (proteintech #20536-1-AP, 1:2,000); mouse anti-Flag M2 antibody (Sigma #F3165, 1:5,000); mouse anti-Myc antibody (Cell Signaling #9B11, 1:5,000); goat anti-mouse conjugated with HRP (Sigma #A4416, 1:5,000); and goat anti-rabbit conjugated with HRP (Thermo Fisher #31460, 1:5,000).

### Image and statistical analyses.

Confocal images for analysis of intracellular localization of SARS-CoV-2 replicase nonstructural proteins were processed using ImageJ or Fiji software. Statistical analysis was performed using GraphPad Prism 8.4.3 software. One-way analysis of variance (ANOVA) was used for multiple comparisons between the protein of interest and other different nonstructural proteins, with a *P* value ≤ 0.05 used as the threshold for statistical significance.
